# Model discovery approach enables noninvasive measurement of intra-tumoral fluid transport in dynamic MRI

**DOI:** 10.1063/5.0190561

**Published:** 2024-04-29

**Authors:** Ryan T. Woodall, Cora C. Esparza, Margarita Gutova, Maosen Wang, Jessica J. Cunningham, Alexander B. Brummer, Caleb A. Stine, Christine C. Brown, Jennifer M. Munson, Russell C. Rockne

**Affiliations:** 1 Division of Mathematical Oncology and Computational Systems Biology, Department of Computational and Quantitative Medicine, Beckman Research Institute, City of Hope National Medical Center, 1500 E Duarte Rd., Duarte, California 91010, USA; 2 Fralin Biomedical Research Institute, Virginia Institute of Technology at Virginia Tech Carilion, Virginia Tech, 4 Riverside Circle, Roanoke, Virginia 24016, USA; 3 Department of Stem Cell Biology and Regenerative Medicine, Beckman Research Institute, City of Hope National Medical Center, 1500 E Duarte Rd., Duarte, California 91010, USA; 4 Department of Physics and Astronomy, College of Charleston, 66 George Street, Charleston, South Carolina 29424, USA; 5 Hematology & Hematopoietic Cell Transplantation, Beckman Research Institute, City of Hope National Medical Center, 1500 E Duarte Rd., Duarte, California 91010, USA; 6 Department of Immuno-Oncology, Beckman Research Institute, City of Hope National Medical Center, 1500 E Duarte Rd., Duarte, California 91010, USA

## Abstract

Dynamic contrast-enhanced magnetic resonance imaging (DCE-MRI) is a routine method to noninvasively quantify perfusion dynamics in tissues. The standard practice for analyzing DCE-MRI data is to fit an ordinary differential equation to each voxel. Recent advances in data science provide an opportunity to move beyond existing methods to obtain more accurate measurements of fluid properties. Here, we developed a localized convolutional function regression that enables simultaneous measurement of interstitial fluid velocity, diffusion, and perfusion in 3D. We validated the method computationally and experimentally, demonstrating accurate measurement of fluid dynamics *in situ* and *in vivo*. Applying the method to human MRIs, we observed tissue-specific differences in fluid dynamics, with an increased fluid velocity in breast cancer as compared to brain cancer. Overall, our method represents an improved strategy for studying interstitial flows and interstitial transport in tumors and patients. We expect that our method will contribute to the better understanding of cancer progression and therapeutic response.

## INTRODUCTION

Interstitial fluid transport is intrinsically linked to the movement of drugs, nutrients, and cells in tissues and is, therefore, especially important for understanding cancer physiology. Cancers in any tissue develop aberrant transport that can affect the movement of drugs into and through tumors. Interstitial fluid flow, in particular, has been identified as a driving force in tumor cell invasion into surrounding healthy tissue.^1–4^ Interstitial flow can also change the surrounding microenvironment, activating fibroblasts,^5,6^ directing immune cell behavior,^7–11^ and inducing angiogenesis.^12,13^ As such, understanding interstitial flows and interstitial transport is vital to understanding disease progression and therapeutic application strategies,^14,15^ though measuring the interstitial fluid flow field noninvasively has remained a challenge, particularly *in situ* and in 3D.

Contrast-enhanced magnetic resonance imaging (MRI) has provided clinical benefit for cancer treatment for many decades.^16^ Given its noninvasive nature and ability to image soft tissues, which are usually difficult to see with other imaging technologies, MRI has become a staple of diagnosing, planning, and providing prognosis in several cancer settings, especially in brain.^17–19^ Importantly, MRI allows acquisition of physiologically relevant information in both space and time, and therefore, represents one of the best methods for studying dynamics *in situ* and in 3D. Over two decades ago, a version of MRI called dynamic contrast-enhanced (DCE-MRI) was developed to allow the study of tissue vasculature.^20^ A DCE-MRI experiment measures signal enhancement due to the presence of an intravenously injected paramagnetic contrast agent in the region of interest. In practice, a series of images are acquired over time, capturing the signals before, during, and after the contrast agent reaches the target tissue, allowing for both visualization and quantification of vasculature in space and in time. Thus, DCE-MRI offers an improved strategy for studying interstitial flows and interstitial transport.

Existing data processing approaches for physical interpretation of contrast enhancement data from DCE-MRI use ordinary differential equation (ODE) models applied to individual voxels, though this method largely fails to incorporate the rich spatial data provided by the modality.^21^ Few methods exist for quantifying the spatially varying effective diffusion coefficient of contrast agent in DCE-MRI,^22,23^ and similarly few attempt to quantify and investigate interstitial fluid flow (i.e., advection) within the tumor.^24–27^ While there do exist MRI sequences which directly measure interstitial fluid velocity, they struggle with separating vascular flow from interstitial flow and require additional imaging sequences to be applied, significantly increasing the time a patient spends in the scanner.^28,29^ Given that DCE-MRI is becoming a standard clinical practice and novel methods for its acquisition are being actively developed,^30^ there is growing opportunity to utilize it for studying interstitial flow, without the need for requiring more time in the scanner.

**FIG. 1.**
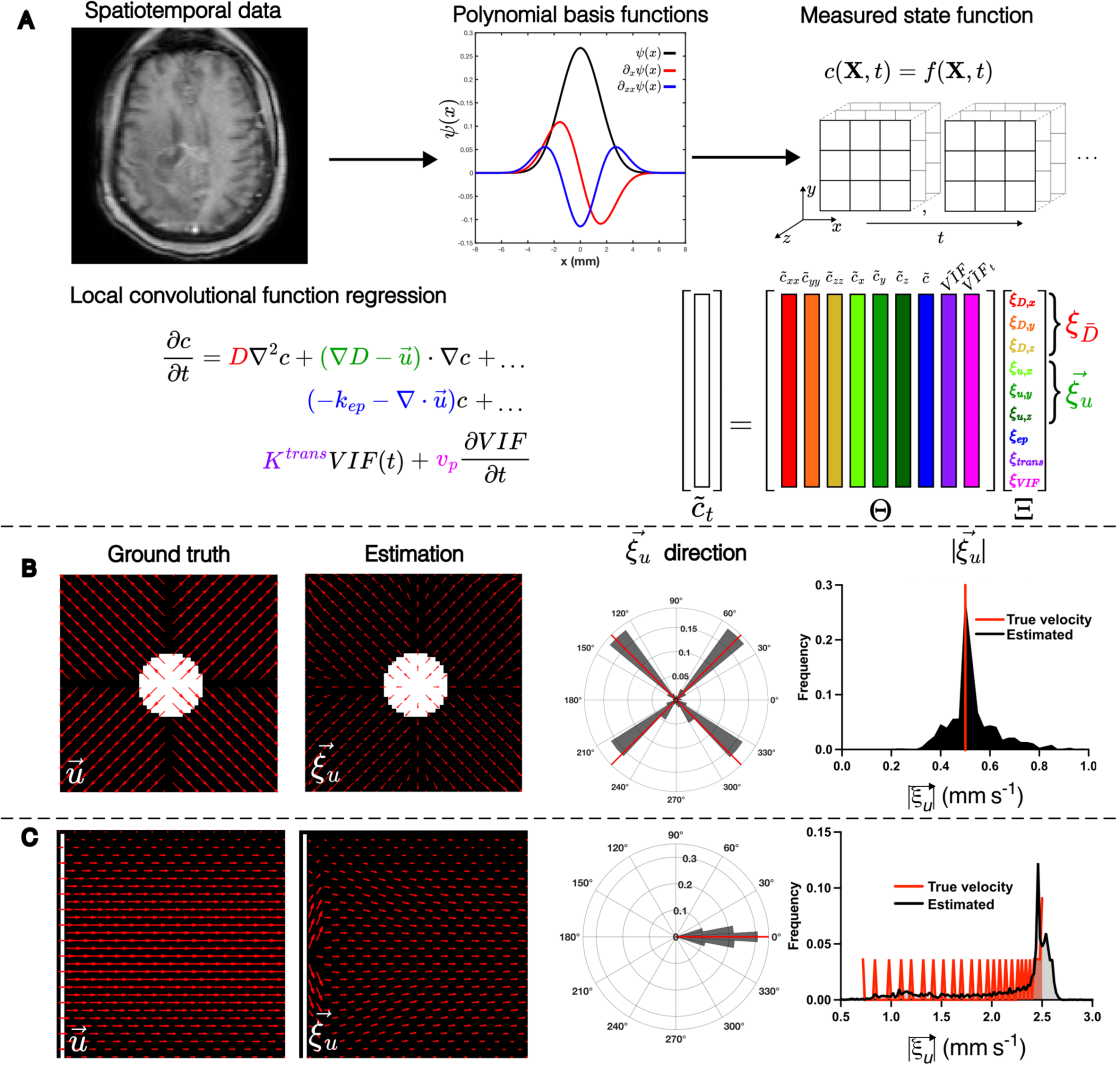
LCFR methodology and validation with *in silico* phantoms. (a) Methodology of localized convolutional function regression (LCFR), wherein spatiotemporal contrast agent concentration data are convolved with a smooth basis-function and its derivatives, divided into 3 × 3 × 3 (x, y, z) windows. The coefficients of the factored transport PDE are then solved for using linear regression. (b) Validation of LCFR coefficient $\vec{\xi_{u}}$ on a divergent flow field (initial condition, white) with spatially invariant diffusion and the true direction and magnitude of velocity denoted in red bars. (c) Validation of LCFR on a Poiseuille shear flow field with spatially invariant diffusion (initial condition, white) and the true direction and magnitude of velocity denoted in red bars.

Here, we develop a strategy that overcomes these limitations by integrating DCE-MRI with a method developed for the discovery of equations governing time-series data through the concept of function-space regression, known in data sciences as sparse identification of nonlinear dynamics (SINDy).^31^ Given that the time- and space-resolved data obtained by DCE-MRI is often noisy,^32^ we utilized a variation of SINDy which leverages the weak form of governing equations to provide an efficient and accurate method for parameter estimation from noisy data.^33,34^ Weak-form methods bypass the use of discrete approximations of derivatives on noisy or sparse data, which are used by the original SINDy implementation^31^ or gradient descent methods used in Jacobian estimation for standard ODE-fitting techniques.^24–26^ The key insight from weak-form methods is the integration of raw data with known basis functions and their derivatives, selected for the problem at hand.^33,34^ However, existing weak-form methods recover global partial differential equation (PDE) coefficients and do not recover spatially varying parameter fields, including IFF. One variation of SINDy, SINDy for boundary value problems, recovers spatially varying PDE coefficients;^35^ however, it does not utilize the weak form of the PDE. In the present study, we apply weak-form function regression on localized patches in the MRI region of interest, recovering a spatially varying field of PDE coefficients from noisy data. We abbreviate this method as localized convolutional function regression (LCFR). We assumed that the dynamics of contrast agent transport are governed by an advection-diffusion-reaction PDE, with vascular input forcing function (VIF), and an unmixed enhancing plasma-compartment, consistent with extended Tofts–Kety intra-voxel transport, advective and diffusive inter-voxel transport [Equation (1), Fig. 1(a)]. These model terms are then estimated as the coefficients, obtained by regression with the function library in Equation (2). This method allows for using domain-specific knowledge to constrain the function library and inform its physical interpretation, and rapid, accurate recovery of spatially varying PDE coefficients within the original image context, and does so efficiently with simple regression. We validate the method on simulated data as well as experimentally in a hydrogel model. Furthermore, we demonstrate that our method works well with data obtained *in vivo* using a mouse xenograft model and show the clinical potential by examining fluid dynamics in human glioblastoma brain tumors and breast cancer datasets.^36,37^ Our approach to DCE-MRI data processing offers a fast and accurate strategy for studying interstitial flows and general fluid transport in space and time *in vivo*.

### Localized convolutional function regression

We designed the parameter estimation method to handle the partial differential equations (PDEs) relevant to DCE-MRI, for performing localized convolutional function regression to recover spatially varying fluid transport parameters [Fig. 1(a)]. Briefly, we assumed that the dynamics of contrast agent transport are governed by an advection-diffusion-reaction PDE, with vascular input forcing function (VIF), and an unmixed enhancing plasma-compartment ( $v_{p}$), consistent with extended Tofts–Kety intra-voxel transport ( $K^{\text{trans}},\;K^{ep}$), advective ( $\vec{u}$) and diffusive ( D) inter-voxel transport:

$$\partial_{t}c=\nabla \cdot (D\nabla c)-\nabla \cdot \left(\vec{u}\cdot c\right)+K^{\text{trans}}VIF-K^{ep}c+v_{p}\partial_{t}VIF.$$ (1)

A mapping between the coefficients and the terms of the full expansion of equation (1) can be found in the supplementary material. We then projected the concentration of contrast agent, c(X,t), onto smooth, 4D polynomial basis functions Ψ(X,t), with compact support in *x*, *y*, *z*, and *t* (supplementary material, Fig. S4). After the data are projected onto basis functions, we perform a spatially localized regression of the following form:

$${\tilde{c}}_{t}=\Theta \left(c\left(X,t\right),\;\mathrm{VIF}\left(X,t\right)\right)\;\Xi ,$$ (2)

where $\tilde{\cdot }$ indicates convolution of the data onto basis functions or their derivatives, denoted as $\tilde{f}=\langle f\left(X,t\right),\Psi (X,t)\rangle$ and ${\tilde{f}}_{q}=-\langle f\left(X,t\right),\partial_{q}\Psi (X,t)\rangle$, respectively [supplementary material, Eqs. (21) and (22)] The model regression problem is performed on each voxel in the region of interest by sampling the weak derivatives in the function library at each (x, y, z) 3 × 3 × 3 window centered at that voxel, and at all time points. The matrix $\Theta \left(c\left(X,t\right),\;\mathrm{VIF}\left(X,t\right)\right)$ is populated by sampling the polynomial projections of the data at each measurement point in the 3 × 3 × 3 window, forming the columns of $\Theta =[{\tilde{c}}_{xx},{\tilde{c}}_{yy},{\tilde{c}}_{zz},{\tilde{c}}_{x},{\tilde{c}}_{y},{\tilde{c}}_{z},\tilde{c},\tilde{\mathrm{VIF}},{\tilde{\mathrm{VIF}}}_{t}]$. The model regression problem is solved locally in space, independently on each 3 × 3 × 3 pixel window, over all time, to recover the spatially localized vector Ξ, which consists of coefficients of the governing equation (1) at each position in space as $\Xi ={\left[\xi_{D,x},\;\xi_{D,y},\;\xi_{D,z},\;\xi_{u,x},\;\xi_{u,y},\;\xi_{u,z},\;\xi_{ep},\xi_{\text{trans}},\xi_{\mathrm{VIF}}\right]}^{T}$. Details of test function construction and hyperparameter selection may be found in the supplementary material.

This method allows for using domain-specific knowledge to constrain the function library and inform its physical interpretation, and rapid, accurate recovery of spatially varying PDE coefficients within the original image context, and does so efficiently with simple regression. Below, we assess the accuracy of this method using multiple examples.

## RESULTS

### LCFR accurately estimates transport parameters from simulated data

To validate our approach, we first examined whether our methodology can recover local PDE coefficients *in silico* from forward model simulations. An advection-diffusion model was used to generate two flow patterns (Table I). The first model we considered is a radially diverging flow field [Fig. 1(b)], which was chosen because it simulates the outward flow typically expected of a high-pressure tumor and will test the method's ability to distinguish advective and diffusive dispersion. The second model we implemented was a Poiseuille shear flow [Fig. 1(c)], a standard case of incompressible flow in a pipe or blood vessel, with a well-studied spatially varying velocity field. In both cases, LCFR accurately recovers the direction and magnitude of the flow field after addition of 0.1% noise (% maximum signal), with root mean squared error (RMSE) of 1.02 × 10^−1^ mm/s for divergent flow, and RMSE = 4.63 × 10^−1^ mm/s for Poiseuille flow. To study the relationship between error and noise, we repeated this analysis with the addition of noise at multiple levels. For the outward flow scenario [Fig. 1(b)], the median error in velocity was 39.8% at 10% noise, 11.2% error at 1% noise, and less than 10% error at noise levels less than 1% (supplementary material, Fig. 9). For the Pouiselle flow scenario [Fig. 1(c)], the median error in velocity was 52.3% at 10% noise and converged to 10.1% error at noise levels less than 1% (supplementary material, Fig. 9). Full parameter error convergence plots for all computational scenarios may been seen in the supplementary material, Fig. 9. Of note, we removed edge effects due to the Gibbs phenomenon from the visualization and analysis.

**TABLE I.** Description of *in silico* simulation parameters.

Simulation	Diffusion coefficient	Velocity field	Source dynamics	Explicit FDM time step (dt)
Divergent flow	0.75 mm^2^ s^–1^	$u_{x}=\frac{\sqrt{2}}{2}\;\text{sign}(x)$ mm s^−1^ $u_{y}=\frac{\sqrt{2}}{2}\;\text{sign}(y)$ mm s^−1^	None	0.125 s
Poiseuille shear	0.75 mm^2^ s^–1^	$u_{y}=0$ mm s^–1^ $u_{x}=2.5\frac{{\left(1-y\right)}^{2}}{32^{2}}$ mm s^−1^	None	0.125 s
Extended Tofts-Kety	0	0	$K^{\text{trans}}$(1/s): Quadrant 1 = 0.1 Quadrant 2 = 0.2Quadrant 3 = 0.4Quadrant 4 = 1 $v_{e}$ = 0.5 $v_{p}$ = 0.05	3.75 s

To test the ability of the method to identify the transport rate constant $K^{\text{trans}}$ and vascular volume fraction $v_{p}$, we simulated the extended Tofts–Kety dynamics with spatially varying perfusion, $K^{\text{trans}}$, and constant vascular volume fraction, $v_{p}$ = 5.00 × 10^−2^, and 0.1% noise. In this scenario, $K^{\text{trans}}$ was accurately estimated by the coefficient $\xi_{\text{trans}}$ (RMSE = 1.54 × 10^−1^ 1/s), while the measurement of the plasma volume fraction $v_{p}$ (RMSE = 3.89 × 10^−1^) was observed to be influenced by the transport rate $K^{\text{trans}}$ (see the supplementary material). We also compare our method against other methods in the literature for accuracy in estimation of *K^trans^*. At 5% noise, across 100 instantiations of noise, the median relative error in *K^trans^* was measured to be 17.2% (IQR 11.6%–23.8%). A comparison of computational walltime, hardware, and accuracy are summarized in Table II. It is important to note that the most comparable methods are the finite element PDE^22^ and PINN^38^ methodologies, but neither of these methodologies incorporate advective transport. Taken together, our *in silico* validation demonstrates the ability of our method to accurately capture both the direction and magnitude of different fluid velocity fields. We showed that sharp changes in the field can be smoothed out by the basis functions, though the resulting fields are consistent with the true velocity fields. We also demonstrate measurement of Tofts–Kety-like dynamics, wherein $\xi_{\text{trans}}$ accurately estimates *K^trans^*. This gave us confidence to further validate LCFR *in vitro*, *in vivo*, and using clinical data.

**TABLE II.** Comparison of *K^trans^* measurement accuracy and walltime across inversion methods.

Inversion method	Accuracy in *K^trans^* ( $\sigma_{\mathrm{SNR}}=5\%$)	Walltime per subject	Hardware used	Source
LCFR	17.2% (median, computational domain) (present work)	1.5 min (3D volume, mouse subject, 128 × 128 × 12 voxels, 50 time points) (present work)	2.3 GHz 8-Core Intel Core i9, 32 GB 2667 MHz DDR4 RAM (present work)	Present work
Voxel-wise Extended Tofts-Kety ODE Inversion	18.9% (median)^22^ 55% (mean)^38^	101 min (3D volume, mouse subject, 128 × 128 × 12 voxels, 50 time points) (present work)	2.3 GHz 8-Core Intel Core i9, 32 GB 2667 MHz DDR4 RAM (present work)	Sainz-DeMena *et al.*,^22^van Herten *et al.*,^38^Present Work
Finite element PDE Inversion	23.9% (median)^39^	10 h (2D computational domain, 360 time points on 955 nodes)^22^	24 CPUs and 32GB RAM^22^	Sainz-deMena *et al.*,^39^Sainz-deMena *et al.*^22^
PINN	6.3% (median)^39^	30 min (2D computational domain, 60 points in space, 360 points in time)^39^	NVIDIA RTX 3070 GPU, 32 GB RAM, and Intel i7-11700K CPU^39^	Sainz-de Mena *et al.*^39^

### LCFR accurately measures the mean contrast velocity in porous hydrogel

**FIG. 2.**
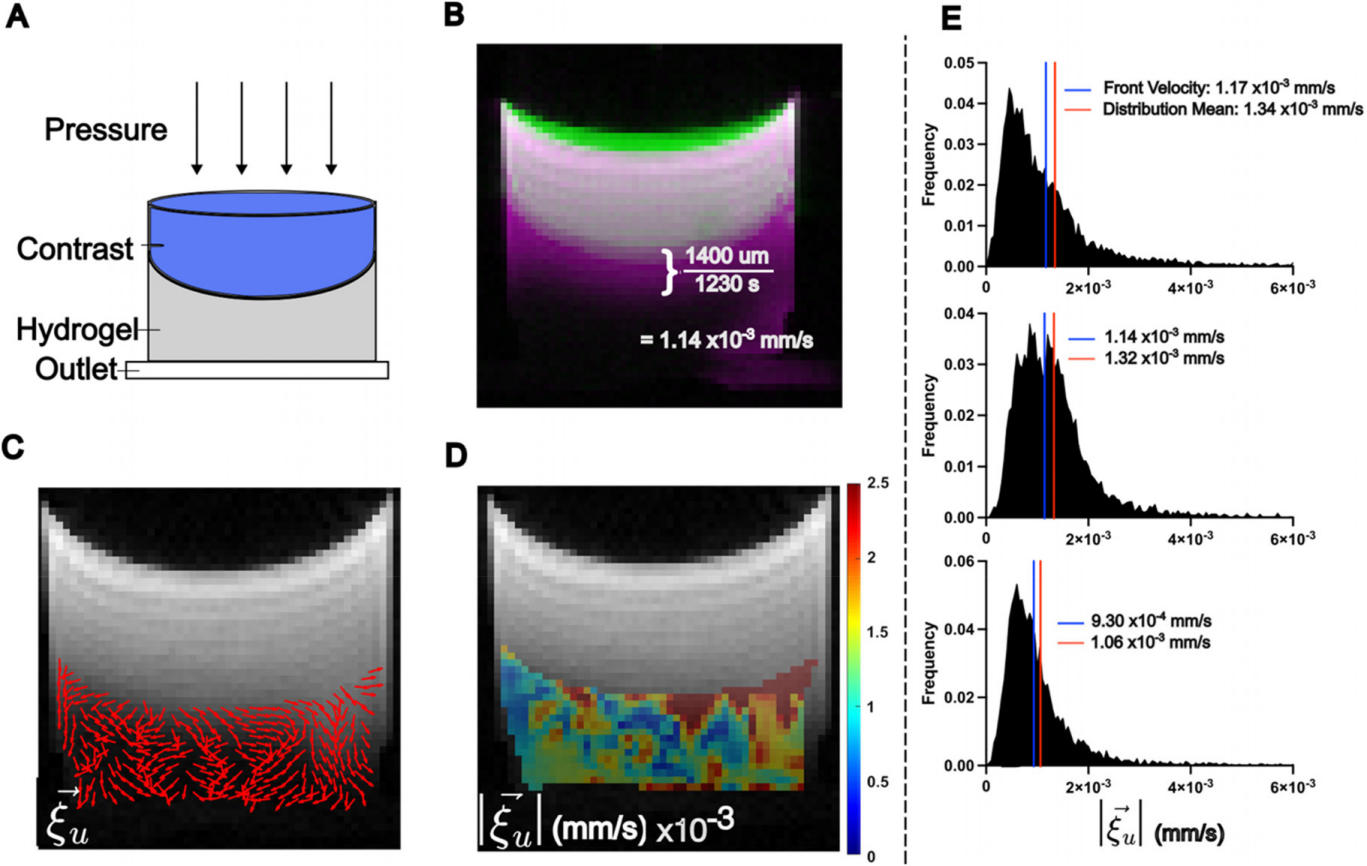
LCFR predicts interstitial fluid velocity in hydrogel phantoms. (a) Experimental setup, wherein a bolus of contrast agent is administered onto a porous hydrogel and drains through due to a hydraulic pressure head. (b) Method of estimating the mean flow velocity of the contrast agent front, using the difference between the initial contrast location (green), and final contrast location (pink), resulting in an estimated contrast agent velocity of 1.14 × 10^−3^ mm/s. (c) The estimated in-plane interstitial flow velocity direction overlaid on the final T1-weighted image. (d) The local 3D magnitude of interstitial flow velocity within the hydrogel. (e) Histograms of three replicate gels, comparing the 3D magnitude of flow velocity within the hydrogel, with the blue line indicating the estimated contrast front velocity, and the red line indicating the mean velocity of the distribution as measured by LCFR.

We next validated the methodology *in vitro*, by administering a bolus of contrast agent on top of porous hydrogel with a pressure head forcing the contrast agent through the gel [Fig. 2(a)]. DCE-MRI was acquired at 30 s time intervals, and signal intensity was converted to contrast agent concentration using T1-mapping. LCFR was applied to the resulting concentration field. We manually estimated the velocity of the contrast agent front ( $\left|\vec{u}\right|=$ 1.14 × 10^−3^ mm/s) [Fig. 2(b)] and compared it to the LCFR-estimated 3D velocity $\left|\vec{\xi_{u}}\right|$ within the hydrogel ( $|\vec{\xi_{u}}|=$ 1.32 × 10^−3^ ± 8.20 × 10^−4^ mm/s) [Figs. 2(b)–2(e)]. The mean measured diffusivity within the gel $\xi_{\bar{D}}$ was measured to be $\xi_{\bar{D}}=$ 1.48 × 10^−4^ ± 7.35 × 10^−6^ mm^2^/s (N = 3). This analysis was performed on multiple replicates, and the mean error associated with the method was 15.2% ± 1.08% (N = 3) [Fig. 2(e)]. Overall, these results illustrate that using LCFR results in accurate estimation of 3D velocity and diffusivity in a controlled system, where there are reliable methods for estimating the true velocity rate and literature-characterized diffusivity. From these findings, we began investigating the results of our methodology *in vivo*.

### Evans blue leakage corresponds to elevated LCFR-measured perfusion kinetics in a murine glioma model

**FIG. 3.**
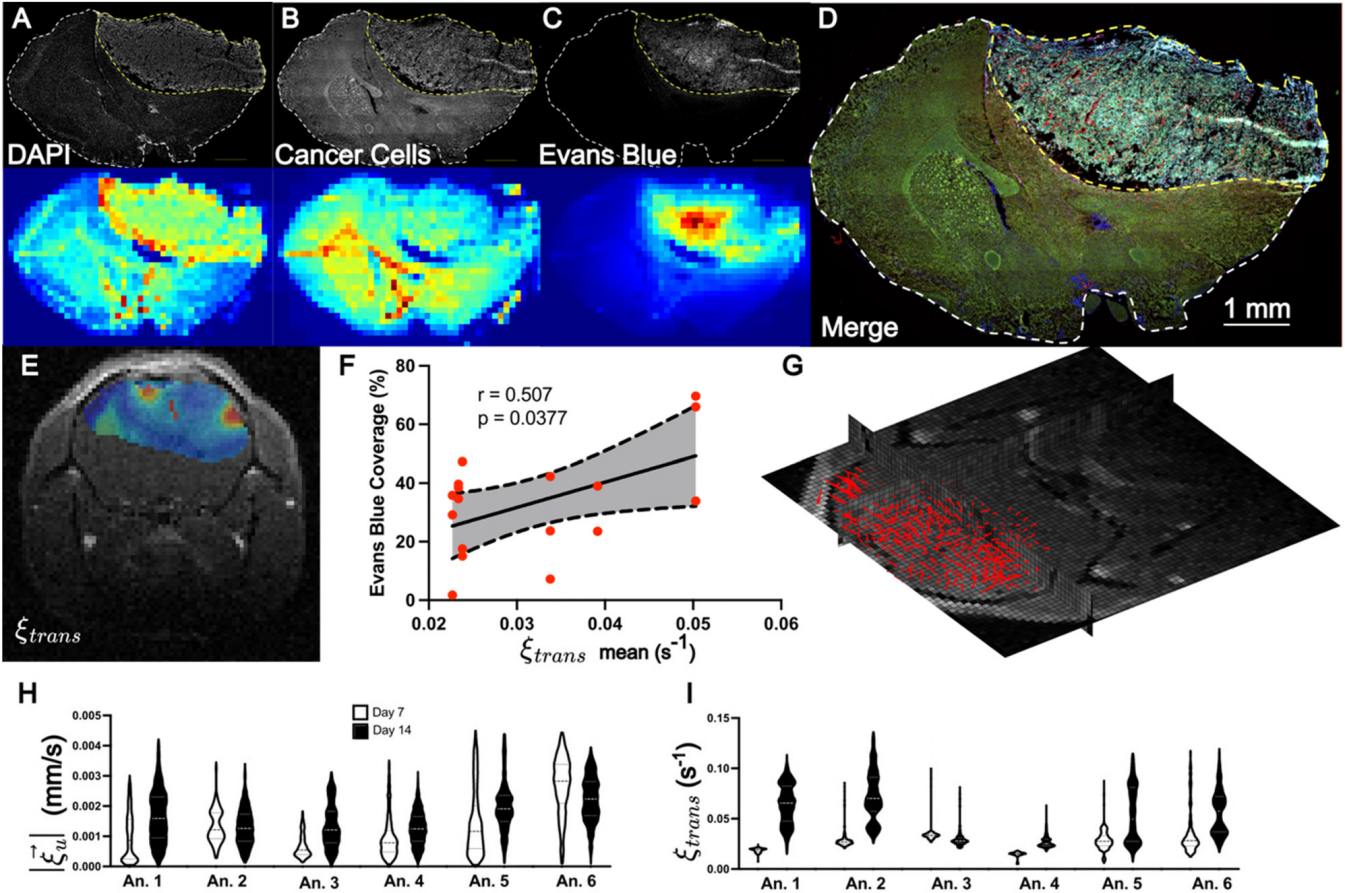
LCFR-measured perfusion is correlated with Evans Blue coverage *in vivo*. (a)–(d) Representative coronal IHC stains through the central tumor slice (top row) and MR resolution-matching intensity projection (bottom row), consisting of DAPI (a), GFP-expressing GL261 cells (b), Evans Blue (c). (d) Merge of all IHC demonstrating tumor heterogeneity. (e) Estimated perfusion field, $\xi_{\text{trans}}$, overlaid on post-contrast T1-weighted image. (f) Scatter plot depicting the correlation and 95% confidence interval of linear regression between mean tumor perfusion as measured by DCE-MRI ( $\xi_{\text{trans}}$) and Evans Blue Coverage (mean tumor stain intensity), for N= 17 histology slices. (g) 3D velocity vector field of estimated interstitial velocity $\vec{\xi_{u}}$, overlaid on the 3D T1 post-contrast volume. (h) and (i) Violin plots depicting the estimated velocity magnitude (h) and perfusion (i) for six animals imaged 7 and 14 days after tumor implantation.

Encouraged by the results from *in silico* and *in vitro* analysis, we subsequently validated the method *in vivo*. Six mice (N = 6) implanted with a mouse glioma cell line (GFP-GL261) in the brain underwent DCE-MRI with isometric spatial resolution of 0.2 mm, at 7 and 14 days post-implantation. After imaging on day 14, the mice were injected with Evans Blue to quantify perfusion, and brains were harvested and stained for visual comparison to LCFR outputs [Figs. 3(a)–3(d)]. For comparison to histology, the estimated perfusion $\xi_{\text{trans}}$ is overlaid on the central tumor slice of the T1-weighted image [Fig. 3(e)]. The mean value of $\xi_{\text{trans}}$ was then compared to the mean area coverage of Evans Blue after venous infusion, and is observed to be positively correlated with the mean tumor perfusion rate constant, $\xi_{\text{trans}}$, across all mice (r = 0.507, P = 0.038, N = 17, two-tailed Pearson correlation) [Fig. 3(f)]. The resulting 3D interstitial velocity field, $\vec{\xi_{u}}$, is displayed over the native post-contrast T1 weighted volume, highlighting that LCFR is readily applied on 4D data [Fig. 3(g)]. The measured velocity $\left|\vec{\xi_{u}}\right|$ remained constant from $\left|\vec{\xi_{u}}\right|=$ 1.35 × 10^−3^ ± 7.04 × 10^−4^ mm/s on day 7 to $\left|\vec{\xi_{u}}\right|=$ 1.63 × 10^−3^ ± 4.04 × 10^−4^ mm/s on day 14 (P = 0.218, N = 6, two-tailed Wilcoxon test) [Fig. 3(h)]. The fluid rate transfer constant, $\xi_{\text{trans}}$, increased from 2.70 × 10^−2^ ± 8.65 × 10^−3^ 1/s on day 7 to 5.08 × 10^−2^ ± 1.84 × 10^−2^ 1/s on day 14 (P = 0.063, N = 6, two-tailed Wilcoxon test) [Fig. 3(1)]. These results demonstrate that LCFR can noninvasively detect individual variation of perfusion and flow, as validated with histology.

### LCFR-measured interstitial fluid velocity varies between breast and brain cancers

**FIG. 4.**
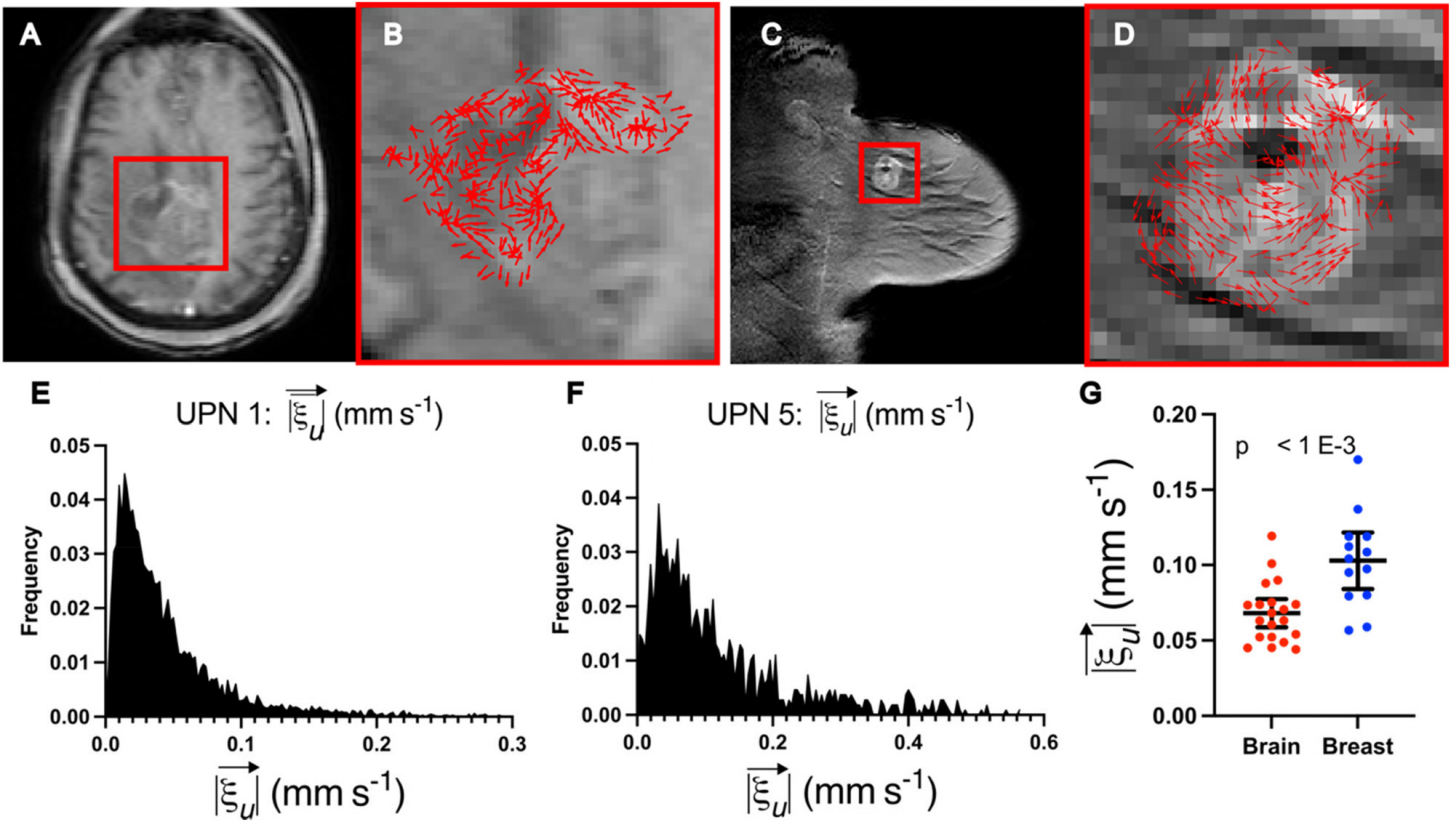
LCFR captures differences in fluid transport between breast and brain cancers. (a) and (b) Representative post-contrast T_1_-weighted image of central slice of residual glioblastoma 2 weeks after resection surgery. (b) Detail of tumor and resection cavity, with overlay of the estimated velocity direction, $\vec{\xi_{u}}$. (c) and (d) Representative post-contrast T_1_-weighted image of untreated primary breast cancer lesion. (d) Detail of the enhancing tumor, with overlay of the with overlay of the estimated velocity direction, $\vec{\xi_{u}}$. (e) Distribution of the in-plane velocity of the entire enhancing glioblastoma and resection cavity (mean = 5.23 × 10^−1^ ± 5.10 × 10^−1^). (f) Distribution of the in-plane velocity of the entire enhancing breast tumor (mean = 1.19 × 10^−1^ ± 1.06 × 10^−1^). (g) Mean fluid velocities for brain (mean = 6.81 × 10^−2^ ± 1.99 × 10^−2^ mm/s, N = 20) and breast data (mean = 1.03 × 10^−1^ ± 3.10 × 10^−2^, N = 13), with whiskers indicating mean and 95% confidence interval.

To investigate the differences in interstitial fluid flow between cancers in different tissues, we applied LCFR to clinical DCE-MRI in a cohort of post-resection treatment naïve glioblastoma patients and a cohort of treatment-naïve breast cancer patients. In the glioblastoma cohort, 20 patients with recurrent disease who underwent DCE-MRI imaging between January 2020 and July 2022 were selected from the radiology records at City of Hope National Medical Center. The glioblastoma patients underwent DCE-MRI on average 32.3 days after resection and prior to receiving additional therapy. The breast cancer cohort consisted of 13 treatment-naïve patients from the Quantitative Imaging Network BREAST-02 study.^37^ In each dataset, the enhancing lesion was manually segmented, and LCFR was run to investigate the population fluid dynamical profile. In these cohorts, we measured the flow velocity, $\left|\vec{\xi_{u}}\right|$, in breast tumors (1.03 × 10^−1^ ± 3.10 × 10^−2^mm/s) to be significantly faster than that in brain tumors (6.81 × 10^−2^ ± 1.99 × 10^−2^ mm/s) (P < 0.001, N_brain_ = 20, N_breast_ = 13, and two-tailed Mann–Whitney test). A representative patient from both breast and brain cohorts as well as a statistical comparison between these two groups may be found in Fig. 4. These preliminary results indicate that cancers of different organs and cellular origins may present with different flow profiles and may provide novel methods for explaining differences in disease progression and treatment response. These results warrant further exploration, which is outside the scope of this initial reporting of our novel methodology.

## DISCUSSION

Interstitial fluid transport plays a key role in many processes connected with cancer physiology, tumor microenvironment, tumor immune response, as well as the response of cancer to treatment. However, physical and physiological properties of interstitial flows and transport remain difficult to study and understand. Here, we describe a novel methodology for analyzing DCE-MRI data, which allows for accurate, noninvasive measurement of fluid dynamics in living tissues. The methodology leverages the rich spatial and temporal data provided by DCE-MRI to enhance our understanding of tissue fluid dynamics. In prior studies, DCE-MRI-derived imaging biomarkers, including *K^trans^* and *K^ep^*, have been shown to be prognostic and diagnostic and are used for inputs to predictive models.^40,41^ By refining our understanding of perfusion and fluid transport within tumors to include interstitial transport, we expect to provide additional clinical benefit beyond standard kinetic parameters.

We have validated our approach using synthetic and experimental data, both *in vitro* by following the flow of contrast agent in a hydrogel and *in vivo* by using a mouse model of glioblastoma. We also demonstrate application of LCFR to human MRI data routinely collected in the clinic from patients with either breast or brain cancer.

The present methodology measured the mean interstitial fluid velocity of breast tumors to be 1.03 × 10^−1^ ± 3.10 × 10^−2^ mm/s vs 6.81 × 10^−2^ ± 1.99 × 10^−2^ mm/s as measured in brain tumors (Fig. 4). It is commonly assumed in the literature that interstitial fluid velocity is governed by Darcy's Law:^42^ $\vec{u}=-K\cdot \nabla p$, where the fluid velocity, $\vec{u}$, is proportional to the gradient of fluid pressure, *p*, by the hydraulic conductivity of the tissue, *K. K* is thought positively correlated with tissue apparent diffusion coefficient of water (ADC).^43^ As the ADC of healthy breast tissue (1.36 ± 0.16 × 10^−2^ mm^2^/s^44^) is higher than healthy ADC of brain tissue (0.84 ± 0.11 × 10^−3^ mm^2^/s^45^), it is reasonable to hypothesize that this difference between the two organs explains the difference in fluid velocity measured by our method. Furthermore, as restricted velocity can result from denser tumors, fluid velocity may be a parallel way to measure individual-specific tumor properties and may be used in the future as a prognostic or diagnostic feature to noninvasively grade tumor aggression.

LCFR was able to accurately measure the mean 3D flow velocity of contrast agent forced through a hydrogel, and yielded mean diffusivity in the gel, $\xi_{\bar{D}}$, consistent with diffusivity measurements reported previously.^46,47^ However, if we compare our results obtained using LCFR to process DCE-MRI mouse data (1.35 × 10^−3^ ± 7.04 × 10^−4^ mm/s) with results from recent studies that used phase-contrast imaging to directly measure fluid velocity within tumors (1.10 × 10^−1^ ± 5.5 × 10^−4^ and 1.10 × 10^−1^ ± 5.5 × 10^−4^ mm/s),^28,29^ we find some discrepancy, likely due to contribution from vascular flow, which could not be disambiguated from tissue interstitial fluid flow in the phase-contrast methods resulting in higher values.^24,25,36,37^ In the present study, the bolus arrival time is corrected for in each voxel, thus minimizing the contribution of vascular velocity in the total velocity field. Additionally, we demonstrate that the spatial gradient of diffusion is structurally unidentifiable from true advective transport and may, thus, artificially increase the apparent velocities observed in past studies and others directly measuring advective transport (supplementary material). These confounding factors may contribute to higher apparent velocities measured by methods which utilize higher temporal resolution data or do not account for diffusive and advective transport separately, thus explaining the observed discrepancy.

Our methodology has several advantages over traditional model inversion methods used to parameterize DCE-MRI data, foremost that it is readily applied in native 4D, instead of individual z-slices, allowing for simultaneous estimation of both inter- and intra-voxel fluid transport parameters. Moreover, LCFR is able to handle noise due to the use of smooth polynomial basis functions and performs similarly to other model inversion methods for DCE-MRI, and is computationally more efficient (Table II). Our method is highly efficient, utilizing the fast Fourier Transform for convolution with basis functions and linear regression for the recovery of local PDE coefficients. Finally, our method allows for a direct calculation of parameters of a PDE from the original data, agnostic of spatial and temporal resolutions, and without the need for iterative forward-PDE solutions required for PDE inverse problems.^48,49^

While these advantages are useful for this type of data, they do come with tradeoffs which limit the performance of the method. For example, our method uses a pre-defined library of functions to characterize the dynamics ( Θ), instead of an extensive library of hypothetical functions and polynomial combinations of partial derivatives. This is largely because neither the higher-order and spatial cross-derivatives, nor products of these terms are readily interpretable with respect to the physics at hand, and storage of each of these 4D arrays may be memory-expensive. Additionally, we utilize L2 regression for simplicity and efficiency, as opposed to sparse objective functions such as LASSO or SR3, which are typically used in model discovery frameworks.^34,50^ For this reason, we refer to our method as function regression, instead of model discovery, as it utilizes a set library constructed from prior knowledge of the physics problem at hand. Some of the PDE parameters are unidentifiable given the structure of the underlying PDE and characteristics of the vascular input function. Furthermore, due to the nature of any overdetermined regression problem, the models recovered may not be unique. This is especially the case for model discovery methods which utilize L1 or SR3, as the discovered model strongly depends on the sparsity parameters used to enforce parsimony. While the present methods may not yield unique results for each individual spatial window, these results consistently indicate the presence of directed transport within tumors and allow for the accurate measurement of transport parameters *in vitro* and within living tissue.

## CONCLUSION

In this work, we present a data processing framework tailored specifically to the unique needs of DCE-MRI for studying fluid dynamics in living tissues. These developments were fueled by our interest in understanding interstitial fluid flow and transport, as a key contributing factor influencing cancer biology, progression, and response to therapy. Given the lack of tools in this area, our contribution will be of significant interest to the community. In our strategy, we employ approaches from data science and integrate them into DCE-MRI data analysis framework, which allowed us to process and analyze noisy data rapidly *in situ*. Importantly, in an *in vivo* mouse study, we noninvasively capture individual variation in tumor perfusion and interstitial flow over time and find that perfusion estimated by this noninvasive method are consistent with measures of perfusion and vascular density measured from tissue histology. Finally, we find that fluid velocity magnitude in brain and breast cancers differ significantly, finding the measured velocity to be greater in breast than in brain. These results support use of our method in measuring both variations across individuals and between diseases of different origins, providing a novel method for studying the underlying physiology, and demonstrating the application of this novel methodology to routinely collected clinical imaging.

## METHODS

### In silico forward methods

Forward PDE models of advection, diffusion, and source of contrast in tissue are implemented using the two-dimensional finite difference method on a 64 × 64 mm^2^ grid, with Δx=Δy=1mm, with explicit time stepping. The boundary conditions are Dirichlet such that ( $c{\left(X,t\right)}_{\partial \Omega }=0$). After the simulation is finished, 100 instantiations of noise was applied for each of ten noise levels *N_strength_*, spaced evenly in log10 space from 10^−9^ to 1. The noise is additive and normally distributed with standard deviation $\sigma_{\mathrm{SNR}}=\underset{t}{\text{max}}\left(c\left(t\right)\right)\;N_{\text{strength}}$.

### Collagen-hyaluronic acid hydrogel *in vitro* validation

350 *μ*l rat collagen I (0.2%)-photo-crosslinkable hyaluronic acid (0.4%) was pipetted into a 12 mm tissue culture insert (Millipore, Burlington, MA) and crosslinked for 45 s. Prior to imaging, 100 *μ*l of 1× PBS was applied below the tissue culture insert in a collection chamber. To induce flow through the gel, 300 *μ*l of a 1:100 dilution of Gd-DTPA (BioPal, Worcester, MA) in 1X PBS was administered atop the gel. The contrast front velocity was measured by calculating the number of pixels contrast traveled through the gel during the duration of MR imaging [Fig. 2(a)]. The location of these sample points may be found in supplementary material, Table 1. Dynamic contrast enhanced imaging was performed according to methods for *in vivo* analysis (see Methods: Magnetic Resonance Imaging, 3D DCE). A single T10 map was acquired through VFA methods (see Methods: Magnetic Resonance Imaging, 3D DCE), across three replicates, and the mean T10 value within the gel was used for the T10 value across the three replicates to calculate the concentration of Gd-DTPA within the hydrogel.

### Description of cell lines

GL261-GFP cells were generated as previously described.^3^ Cells were serially transduced with GFP lentivirus and purified by selection with 2 *μ*g/ml puromycin (Thermo Fisher A1113803). Cells were maintained at 37 °C and 5.2% CO_2_ for at least three passages after thaw with DMEM + 10% Fetal bovine serum (ThermoFisher, Gibco). Cells were resuspended at a concentration of 20 000 cells/*μ*l in serum free media for tumor implantation.

### Description of *in vivo* animal studies

Three-month-old, male C57Bl/6 mice (n = 6, ∼25 g) were purchased from Charles River. The animals were housed in a room maintained at 20–23 °C, 45%–55% relative humidity, and a 14-h/12-h light/dark cycle with access to standard laboratory chow and water until the experiment. Mice were anesthetized and connected to a stereotactic frame. A burr hold was drilled at stereotactic coordinates (−1.25, 1.25, −1.91) with respect to lambda. 100 000 GFP-Gl261 (NCI-DTP Cat# Glioma 261, RRID:CVCL_Y003) cells were resuspended in 5 *μ*l and injected via a Hamilton syringe and pump (World Precision Instruments) at 1 *μ*l/min. The syringe was left for three additional minutes following injection completion to prevent reflux. Tumors were imaged with MRI on days 7 and 14 and tissues were harvested on day 15. Following inoculation, mice were provided wet food and hydrogel for recovery, injected with 4 mg/kg ketoprofen for 48 h post inoculation, and weighed every other day for the duration of the study. Mice were allowed to live in group housing, with a max of four mice per cage. Mice were separated if wounds appeared indicating aggressive behavior.

### Small animal magnetic resonance imaging

Following anesthesia, a catheter was inserted into the lateral tail vein. Mice were imaged with a 9.4 T small animal MRI (Bruker, Ettingen, Germany) equipped with a 20 mm RF surface coil. Two consecutive T2-weighted images were collected to verify tumor presence. The first image has high SNR to aid with MRI:IHC alignment for cryosectioning; the second T2-weighted image has isometric voxels for registration to isotropic T1 images. T1 mapping was performed to collect baseline intensity, followed by a 3D DCE T1-weighted FLASH sequence. Six pre-contrast images were acquired before injecting gadolinium (0.2 ml/kg, BioPal). A T1-weighted post-contrast image was acquired to confirm contrast enhancement. Imaging resolutions and fields of view for small animal and hydrogel imaging may be found in Table III. An extensive list of imaging parameters are included in Table S2. After completion of MRI, mice were injected with Evans Blue at a concentration of (1.6 ml/kg) which was allowed to circulate overnight before tissue harvesting.

**TABLE III.** Small animal and hydrogel MRI imaging parameters.

	1. T2-weighted	2. T2-Weighted	3. T1 mapping	4. T1-weighted DCE	5. T1-weighted
Sequence	RARE	RARE	RARE with varied TR	FLASH	FLASH
TE/TR	40/2600 ms	40/2600 ms	TE: 7 msTR: 5500, 3000, 1500, 800, 500, 300 ms	4/21 ms	3/180 ms
Number of slices	16	12	12	1 (3D imaging)	16
Slice thickness (*μ*m)	400	200	200	200	400
Rare factor	8	8	2	n/a	n/a
Flip angle	90,180	90/180	90/180	25	70
FOV (mm^2^)	19.2 × 19.2 mm^2^	19.2 × 19.2 mm^2^	19.2 × 19.2 mm^2^	19.2 × 19.2 × 12 mm^3^	19.2 × 19.2 mm^2^
Matrix size	192 × 192	96 × 96	96 × 96	96 × 96 × 12	192 × 192
Repetitions	1	1	1	48	1
Averages	9	18	18	1	7
Time of acquisition	9 min 21 s	9 min 21 s	9 min 16 s	24 min 11 s	3 min 1 s

### Tissue harvest and immunohistochemistry (IHC)

Mice were euthanized and transcardially perfused with 4% PFA in ice cold 1× PBS. Brains were post-fixed in PFA for 18 h and placed in 30% sucrose until complete submersion. Afterward, brains were placed in molds with O.C.T. Compound at −80 °C and sectioned at 12 *μ*m on a cryostat. T2-weighted MRI images were used as a guide during sectioning to inform which MRI slice corresponded to the collected cryosectioned slice. Structural features (i.e., ventricles, white matter, tumor shape) were used as visual aides to confirm location and compared to the corresponding coronal MRI slice. Brain Secs. were stained for DAPI (ThermoFisher) and imaged at 20× on an VS200 Olympus Slide Scanner (Olympus).

### Immunohistochemistry comparison to MRI

To visually compare MRI to histology, all IHC stains were first loaded into MATLAB, and then down-sampled to 0.2 mm in-plane resolution to match the resolution of isotropic MRI. This down-sampling was done using the *blockproc* function in MATLAB, summing the total intensity of sub-pixels within the larger superpixels so as to maintain the total image intensity. A ROI was drawn around the tumor region of the DAPI stain, using the *drawfreehand* function in MATLAB, as well as on the corresponding slice of post-contrast DCE-MRI.

### Immunohistochemistry Evans Blue coverage calculation

Evans Blue stains from individual animals were processed using FIJI image processing software to calculate percent tumor coverage. First, a threshold was applied, removing the lower 95th percentile of the image intensity. A mask of the tumor was draw on the DAPI stain to demarcate the tumor using FIJI's “mask” functionality. This mask was then applied to the Evans Blue stain, and the percent of voxels greater than the 95th percentile intensity within the tumor ROI was reported. The percent tumor Evans Blue coverage was then taken and compared to the mean tumor intensity value of $\xi_{\text{trans}}$ on an individual animal basis, and the correlation and significance of the correlation were determined using a Pearson correlation test.

### Clinical imaging: QIN BREAST-02

The study of publicly available data were approved by the local Institutional Review Board Protocol 15286. All 13 patients from the QIN BREAST-02 dataset, provided by The Cancer Imaging Archive (TCIA), were analyzed using LCFR. All patients (female, 18+) were diagnosed with invasive breast cancer, with lesion size > 1 cm. Images used were while all patients were treatment-naïve, though all patients received treatment after initial imaging. The multi-flip T1 map and DCE sequences, acquired on Phillips 3 T scanners located at both Vanderbilt University Medical Center and University of Chicago, were used in this study.^37^ Image acquisition details can be found on the TCIA website for the BREAST-02 study.

### Clinical imaging: Glioblastoma patients

The retrospective study of City of Hope patient data was approved by the local Institutional Review Board Protocol 15286. 20 patients who came to City of Hope National Cancer Center in Duarte, CA, for advanced imaging after resection of glioblastoma between January 2020, and July 2022, with residual enhancing lesion size > 1 cm underwent DCE-MRI. Patients underwent imaging between 3 and 135 days post-resection (mean = 32.3, median 28, SD = 30.7). Scans were performed on a 3 T Siemens scanner. The DCE scan consisted of 3D FLASH sequence with prior variable-flip angle T1-mapping. Variable Flip angles were acquired at 2°, 5°, and 10°, repetition time = 9.3 ms, and echo time of 4.29 ms. The dynamic scan was performed with 50 phases at 6 s temporal resolution, flip angle = 15°, repetition time = 9.3 ms, and echo time of 4.29 ms. 8 ml of Gadovist (Bayer, Whippany, NJ) was administered during the sequence. The size of the imaging FOV was 192 × 132 in-plane (1.46 mm resolution) and 16 slices with 5 mm slice thickness, identical between the dynamic scan and variable flip angle scans.

### DCE-MRI pre-processing

Each of the variable flip angle images and dynamic T1-weighted images are rigidly registered to the first dynamic T_1_ image. From images acquired using variable flip angles, α, a T_10_ map for each individual was is calculated by regression:^51^

$$S\left(t\right)=S_{0}\frac{\left(1-\text{exp}\;\left(-\frac{T_{R}}{T_{10}\left(t\right)}\right)\;\right)\text{sin}(\alpha )}{1-\text{cos}\left(\alpha \right)\;\text{exp}\left(-\frac{T_{R}}{T_{10}\left(t\right)}\right)}.$$ (3)

Here, *S* is the measured signal intensity, *S*_0_ is the baseline non-contrast-enhanced signal intensity, *T_R_* is the repetition time, *T*_10_ is the native T_1_ relaxation time of the tissue, and α is the flip angle.

From the calculated *T*_10_ map,^52^ and the relaxivity (r_1_) of the contrast agent (Gadovist, 3.7 s^−1^ mM^−1^ for 3 T, and 3.3 7 s^−1^ mM^−1^for 7 T^53^) the sequential T_1_-weighted images are converted into a spatiotemporal map of contrast agent concentration^54^ by

$$R_{1}=r_{1}C_{t}\left(t\right)+R_{10},\;R_{1}=\frac{1}{T_{1}},$$ (4)

$$\frac{1}{T_{1}(t)}=\frac{1}{T_{10}}+r_{1}C_{t},$$ (5)

where R_1_ is the inverse of the *T*_1_-relaxation time, and R_10_ is the baseline relaxation, or the inverse of the baseline *T*_10_ relaxation time.

After calculation of contrast agent concentration, the local contrast bolus arrival time (BAT) is calculated by bilinear regression.^55^ Briefly, a sub-set of each individual voxel's time-enhancement curve, c_sample_, is considered, from the initial time point to the time point with maximal concentration of contrast agent. The points 0 and t_f_ correspond to the initial and final timepoints, and the point p_1_ corresponds to a time points BAT ϵ (0, t_f_). The BAT is determined to be the value that minimizes the summed square error between the bilinear fit and the data

$$\mathrm{BAT}=\text{argmi}n_{\mathrm{BAT}}\left(\Sigma {\left(f\left(i,\mathrm{BAT}\right)-c_{\text{sample},i}\right)}^{2}\right),$$ (6)

f(i,BAT)=m1t+b1,    t≤BAT,m2t+m1BAT, i>BAT. (7)

An empirical vascular input function (VIF) is then measured using the automatic-VIF selection algorithm detailed by Singh *et al.*^56^ Briefly, this method selects voxels which are rapidly enhancing (BAT < 10 s) and enhance within the upper 90th percentile of all voxels. The signal intensity is then normalized and scaled to account for partial volume effect and hematocrit. This method is utilized for the CoH GBM patients, the QIN BREAST-02 dataset, and *in vivo* mouse model. The individual-specific VIF is adjusted for each individual voxel such that the BAT for the VIF matches the estimated BAT of the individual voxel's enhancement time course, using [Eq. (6) and (7)].

## SUPPLEMENTARY MATERIAL

See the supplementary material for detailed descriptions of the methodology, including mathematical formulation of LCFR, detailed equations, pseudocode, experimental convergence analysis, technical limitations, and full clinical data summary for all individuals included in the presented analysis.

## Data Availability

The data that support the findings of this study are available from the corresponding author upon reasonable request.
